# Tibiotalar arthrodesis for injuries of the talus

**DOI:** 10.4103/0019-5413.38588

**Published:** 2008

**Authors:** Jaswant Singh

**Affiliations:** Singh Orthopedic Hospital and Research Center, Station Road, Kota - 324 001, India

**Keywords:** Anterior tibial sliding graft, arthrodesis, avascular necrosis of talus

## Abstract

**Background:**

Fracture-dislocation of the talus is one of the most severe injuries of the ankle. Opinion varies widely as to the proper treatment of this injury. Since Blair's original description of the tibiotalar fusion in 1943, there is little mention in the literature of his method. The present study reports the results of tibiotalar arthrodesis with modification in Blair's technique.

**Materials and Methods:**

Eleven cases of modified Blair's tibiotalar arthrodesis were retrospectively studied. The average age was 32.4 years (range, 26-51 years). Six patients had posttraumatic avascular necrosis; five had neglected fracture-dislocation of the talus.

**Results:**

The mean followup is 8 years (range 3-12 years). Tibiotalar fusion was achieved in all the ankles at an average of 20.5 weeks (range 16-28 weeks). Nine cases having 15°-20° tibiopedal motion had excellent results and two ankles having 10°-15° of tibiopedal motion had good result.

**Conclusion:**

We achieved good long term results with tibiotalar arthrodesis with modification in Blair technique. The principal modification in the present study is retention of the talar body while performing arthrodesis with anterior sliding graft. The retention of the talar body provides intraoperative stability and in the long term, the retained talar body shares the load transmitted to the anterior and middle subtalar joints thus resulting in improved hind foot function and gait.

## INTRODUCTION

Fracture-dislocation of the talus is one of the most challenging problems particularly if these injuries are neglected or if the primary osteosynthesis has failed; thus resulting in nonunion or avascular necrosis of the body of the talus.

Several treatment options are available to treat such disabling injuries. Detenbeck and Kelly^1^ recommended talectomy and tibiocalcaneal arthrodesis, but it has the disadvantage of widening the hind foot and shortening of the foot. Blair^2^ described tibiotalar fusion with excision of the body of the talus and sliding a cortical bone graft anteriorly between the anterior aspect of the tibia and the head of the talus. Thereafter Morris *et al*.^3^ modified this procedure. The modification involves removing the talar body and stabilizing the calcaneum on the tibia by placing a Steinmann pin through the calcaneum and then a screw is placed in the tibial sliding graft to prevent proximal displacement. Subsequently, Dennis *et al*.^4^ used a similar procedure minus the Steinmann pin. They concluded that the normal appearance of the foot is retained after these modifications.

Most of the authors have advocated resection of the talar body, even though the talar body and subtalar joint shares three to four times loads of body weight during normal walking.^5^ This suggests that the resection of the entire body would substantially change the contact characteristic of the remaining anterior and middle facets, therefore this would increase the likelihood of degenerative arthritis.

The purpose of this paper is to report the end results of the treatment of five cases of neglected fracture-dislocation and six cases of posttraumatic avascular necrosis of the body of the talus by modifying Blair's method of fusion, where the body of the talus is retained.

## MATERIALS AND METHODS

**Figure 1:**
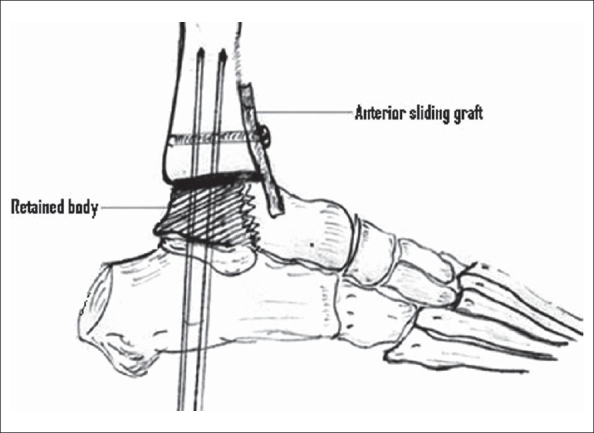
Line diagram of tibiotalar arthrodesis shows preserved osteonecrotic/dislocated talar body. Opposing articular surfaces of tibia and talus are removed; the body of talus is stabilized onto the tibia and calcaneum by K wires. The anterior sliding graft from tibia is placed into the neck of the talus

Between 1994 and 2003 11 tibiotalar arthrodesis by modifying Blair's technique were performed by the author [Figure 1]. The average age was 32.4 years (26-51 years), the M:F. ratio was 4.5:1. Five patients were operated for neglected fracture-dislocation of the talus (the time between injury and arthrodesis ranged from six weeks to 20 weeks with an average of 78 days), the other six for radiographically proven osteonecrosis of the talus. They had been treated in the past either by osteosynthesis (*n* = 4) or by casting (*n* = 2).

### Surgical procedure

**Figure 2:**
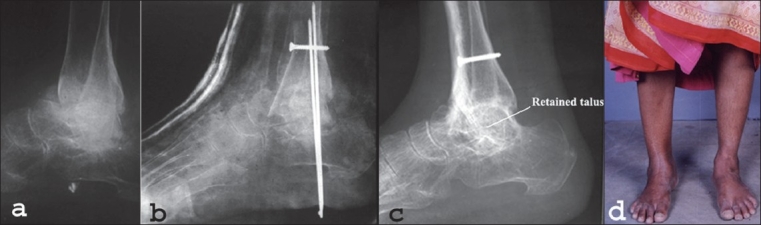
Lateral X-ray (a) of ankle and foot in a 58-year-old woman with eight months' old neglected avascular necrosis of the talus. Immediate postoperative lateral X-ray (b) of the same shows tibiotalar arthrodesis after removal of loose pieces from necrotic talar body and anterior sliding graft from tibial stabilized with transcalcaneal k-wires. Lateral X-ray (c) of ankle of the same patient at 3 years follow-up shows incorporation of tibial and body of talus with cross trabeculation. The clinical photograph (d) at 3 years follow-up shows normal looking appearance

Through the anterior approach the interval between the extensor hallucis longus and extensor digitorum longus is developed with the neurovascular bundle retracted medially. The capsule, periosteum and synovium are incised in line with the skin incision. With the joint widely exposed, the tibial articular surface is denuded and avascular necrotic loose pieces of talar body are removed. The remaining compact talar body is left in place, tibiotalar surfaces are made to firmly contact each other, then anterior sliding arthrodesis is performed. For sliding the graft a 1.5 cm × 6 cm cut is made in the distal anterior portion of the tibia, this graft is snuggly fitted into a slot deeply gouged about 2 cm into the neck of the talus [Figure 2]. The foot is kept in about 5°-10° of plantar flexion while the graft is slid into place. The placement of the graft into the talus and alignment of foot to the ankle and leg are carefully assessed by intraoperative radiographs. Cancellous bone from the tibial graft site is then harvested and packed around the sliding graft; finally, the proximal portion of the graft is secured with a screw.

**Figure 3:**
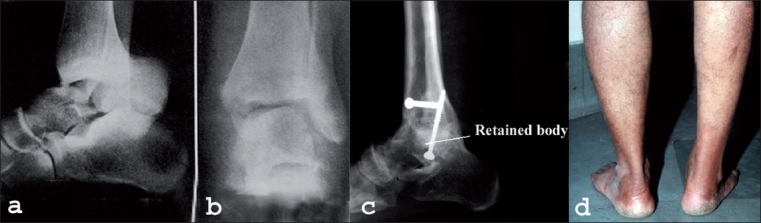
Anteroposterior (a) and Lateral (b) X-ray of ankle and foot of a 30-year-old man having 42 days' old injury shows fracture dislocation of talus. Lateral X-ray (c) nine year of follow-up shows solid fusion. Talar body is incorporated into the tibia, leading to normal hind foot alignment. The clinical photograph (d) at 9 years follow-up shows excellent appearance

In cases of fracture-dislocation of the talus, the best possible anatomic reduction is achieved through both anterior and posteromedial approach; thereafter tibiotalar articular surfaces are denuded and arthrodesis is performed [Figure 3].

Contact between the superior surface of the talus and inferior surface of the tibia must be attained [Figure 1]. Failure to do so may lead to fracture of the graft or nonunion of the arthrodesis. For this reason additional bone grafts from the lower end of the tibia may be harvested. To ensure stability at the site of arthodesis and for prevention of any possible varus or valgus deformity of the foot, additional transcalcaneal k-wires were passed through the calcaneum and retained talar body into the tibia [Figures 1 and 2b]. In one case intraoperative fracture of the graft occurred, hence, external fixator was applied to achieve stability.

Postoperatively long leg plaster was applied for six weeks in the k-wires group and below knee plaster of Paris cast was kept in external fixator group for six weeks. Then after six weeks implants were removed and below knee cast was substituted. The determination of when to put weight on the talus was made after radiographic evidence of healing of the graft.

## RESULTS

The patients with minimum followup period of three years after the arthrodesis were analysed. The average follow-up was eight years (range, three to 12 years). Tibiotalar fusion was achieved in all the ankles at an average of 20.5 weeks (range, 16-28 weeks). The results were considered excellent if the patient was able to return to full activity with a completely asymptomatic foot and ankle. If there was occasional discomfort causing no restriction in activities, then results were considered good; if pain was severe enough to limit activities or to require an analgesic then results were considered poor. By these criteria, results were excellent in nine cases and good in two cases.

The tibiopedal motion is defined as the arc of motion between maximum dorsiflexion and maximum plantar flexion, the angles being those subtended by the long axis of the tibia and that of the foot in the lateral projection. Nine patients, all with excellent results had 15°-20° of tibiopedal motion. Two patients with good result had 10°-15° of range of tibiopedal motion. However, subtalar stiffness was noted in six of the 11 cases. Three of the ankles initially showed restriction of tibiopedal motion and were presenting with the poor results, but when these patients were examined three years after the operation, they all improved their motions, resulting in an excellent result in one and good result in the other two patients.

The position of the fused ankle was assessed clinically in all the patients. Five ankles were at right angle, five had 5° of equinus and one had 10° of equinus.

All the cases were assessed radiologically and there was trabeculation across the retained talus and the tibia. The sliding graft also showed trabeculation across the tibia and talar neck; thus the lower end of the tibia and entire talus became one single block, which was articulating with the navicular anteriorly (talonavicular joint) and with the calcaneum inferiorly (subtalar joint). Limb length discrepancies were also measured and shortening of an average of 0.8 cm was noted.

Gait was assessed and desired compensation in the heel was provided in the footwear. With this modification, the gait improved and no noticeable limp was found. All of them returned to their job with pain-free feet.

## DISCUSSION

The most common and best studied treatment of neglected fracture dislocation and avascular necrosis of talus is the Blair's fusion. Blair^2^ described tibiotalar fusion as primary treatment for fracture of the talus. This procedure involves excising the fragments of the talar body and inserting a sliding tibial graft into the talar neck. Morris *et al*.^3^ and Denis *et al*.^4^ modified this technique of Blair's fusion by stabilizing the graft with a screw and positioning the calcaneus on to the tibia by transcalcaneal steinmann pin. Subsequently, Patterson *et al*.^6^ and Linsy *et al*.^7^ also performed modified Blair's fusion, but none of them tried to salvage the talar body.

There is general consensus, that while performing Blair's fusion, excision of the talar body is mandatory. Kitaoka *et al*.^5^ expressed concern about the talar body removal. They enlightened that when Blair's fusion with excision of the talar body is performed, loads of three to four times body weight occur in the ankle joint during normal walking; similar are the forces on the subtalar joint with removal of the posterior articular facet. The contact characteristic of the anterior and middle facet changes substantially, increasing the likelihood of degenerative arthritis of the subtalar joint. He analyzed 19 patients, by retaining osteonecrosed talus and observed that 16 of the 19 ankles obtained fusion and the talus got incorporated.

Keeping a similar opinion, the author also preserved body of the talus in 11 ankles. The retained talus provided immense intraoperative stability and chances of varus and valgus deviation were minimized. It supported the fusion site like a scaffold and did not allow immediate collapse. This modification also provided larger contact area for the arthrodesis to facilitate union at arthrodesis site. When these patients were analyzed over a period of time (average, eight years), almost normal alignment of the foot relative to the ankle and the leg was observed. Radiologically the talus, the graft and the tibia got incorporated and solid fusion was achieved in all the cases. Hawkins^8^ also observed that the replacement of the body takes several years and proceeds in conjunction with weight-bearing. Dunn *et al*.^9^ also noted revascularization of the talar body; however, it took several years.

It seems that fusion of the ankle has a detrimental effect on the subtalar joint.^10^ On the contrary subtalar stiffness after fusion of the ankle does not necessarily have a detrimental effect on gait. This may be because the axis of the compensatory motion of the midtarsal joint is now parallel to the ankle, while that of the subtalar joint is perpendicular to it.^11^ In the present study, it was observed that six of the 11 ankles showed subtalar stiffness. But at the same time there was compensatory hypermobility at midtarsal joints, suggesting that there was hardly any deleterious effect in spite of subtalar stiffness.

For normal gait 20° of tibiopedal motion is necessary.^12^ It has been emphasized that preservation of talus and limiting arthrodesis at the tibiotalar level preserves hind foot function, beside sharing load to the anterior and middle subtalar joint. This was found to be one of the factors contributing to improving the gait.^5^ In the present series, all the patients showed 10°-20° of tibiopedal motion, resulting in good to excellent gait. The reasons attributed to such improved results after arthrodesis are because of preservation of hind foot function, compensatory hypermobility at midtarsal joint, minimum leg length discrepancy and compensatory heel raise in the footwear.

Mazur^13^ analyzed the gait of 12 arthrodesed ankles and concluded that those ankles which are fixed in more than 10° of plantar flexion have greater limitation of dorsiflexion at midtarsal joints and must either hyperextend the knee if the limb is long, thereby placing the foot flat on the ground or walk on the toes if the leg is short. So such cases that have been fixed in more than 10° of plantar flexion, require rise of their heel in the footwear.

There are many statements in the literature on the best position of fusion. Barr and Record^14^ preferred a position of 5° of equinus; Knight^15^ recommended the right angle as the best position for men. Watson-Jones^16^ recommended 15° of equinus. In the present series, all the patients had either right angle or 5°-10° of equinus. The ankles which were fused in the equinus experienced difficulty in barefoot walking but they improved their gait abnormalities after wearing shoes with the heels of appropriate height to compensate for the plantar-flexed position of the foot.

Although there are many options to fix the arthrodesis and the retained talus, considering the poor osseous quality of the osteonecrotic body, it was difficult to assess which implant is best. Moreover, it was not the objective of the present study to compare types of fixation. However, in the present study the k-wires were preferred over the external fixator.

To conclude, in the current study, the author has preserved the talar body while doing arthrodesis with anterior sliding graft. This modification led to better stability at the arthrodesis. In the long-term follow-up, almost normal looking feet, minimal leg length discrepancy and almost normal gait were achieved.
